# PEEP titration guided by ventilation homogeneity: a feasibility study using electrical impedance tomography

**DOI:** 10.1186/cc8860

**Published:** 2010-01-30

**Authors:** Zhanqi Zhao, Daniel Steinmann, Inéz Frerichs, Josef Guttmann, Knut Möller

**Affiliations:** 1 Department of Biomedical Engineering, Furtwangen University, Jakob-Kienzle-Strasse 17, D-78054 Villingen-Schwenningen, Germany; 2 Department of Anesthesiology and Critical Care Medicine, Section for Experimental Anesthesiology, University Medical Center, Hugstetter Strasse 49, D-79095 Freiburg, Germany; 3 Department of Anesthesiology and Intensive Care Medicine, University Medical Center of Schleswig-Holstein Campus Kiel, Arnold-Heller-Strasse 3, D-24105 Kiel, Germany

## Abstract

**Introduction:**

Lung protective ventilation requires low tidal volume and suitable positive end-expiratory pressure (PEEP). To date, few methods have been accepted for clinical use to set the appropriate PEEP. The aim of this study was to test the feasibility of PEEP titration guided by ventilation homogeneity using the global inhomogeneity (GI) index based on electrical impedance tomography (EIT) images.

**Methods:**

In a retrospective study, 10 anesthetized patients with healthy lungs mechanically ventilated under volume-controlled mode were investigated. Ventilation distribution was monitored by EIT. A standardized incremental PEEP trial (PEEP from 0 to 28 mbar, 2 mbar per step) was conducted. During the PEEP trial, "optimal" PEEP level for each patient was determined when the air was most homogeneously distributed in the lung, indicated by the lowest GI index value. Two published methods for setting PEEP were included for comparison based on the maximum global dynamic compliance and the intra-tidal compliance-volume curve.

**Results:**

No significant differences in the results were observed between the GI index method (12.2 ± 4.6 mbar) and the dynamic compliance method (11.4 ± 2.3 mbar, *P *> 0.6), or between the GI index and the compliance-volume curve method (12.2 ± 4.9 mbar, *P *> 0.6).

**Conclusions:**

According to the results, it is feasible and reasonable to use the GI index to select the PEEP level with respect to ventilation homogeneity. The GI index may provide new insights into the relationship between lung mechanics and tidal volume distribution and may be used to guide ventilator settings.

## Introduction

Under the condition of general anesthesia during mechanical ventilation, patients are sedated and the alveoli in the dependent lung regions may collapse regardless of the recruitment state of the lungs. In the presence of lung injury, such as acute respiratory distress syndrome (ARDS), dependent lung regions are essentially nonaerated, while non-dependent regions remain partially aerated [1]. Under certain conditions both collapse of the dependent regions and overinflation of the non-dependent ones may occur, which may increase the risk of ventilator-induced lung injury [2]. Lung protective ventilation requires low tidal volume and a suitable positive end-expiratory pressure (PEEP) level to minimize ventilator-induced lung injury. PEEP was introduced to maintain the open atelectatic areas and thereby reduce the risk of hypoxemia and cyclic recruitment/derecruitment. Although the application of PEEP is widely used in clinical practice, it remains under debate as to how to titrate the adequate PEEP level for individuals [1]. Increase of PEEP further prevents derecruitment in the dependent areas but may lead to overdistension in the non-dependent areas as well. To find a balance between these two aspects is one goal of setting PEEP.

The information provided by global parameters of lung function, such as blood gas values, dynamic respiratory mechanics indices and slope of the static pressure-volume (P/V) curve does not consider regional inhomogeneity of the lung, and therefore may be sometimes misleading [3].

Computed tomography (CT) has a very good spatial resolution [4] and is able to show the distribution of the tissue density in the chest, thereby providing primarily morphological data. Unfortunately, its application for bedside monitoring is limited due to radiation exposure of patients and complex handling (e.g. large equipment).

Electrical impedance tomography (EIT), as a noninvasive and radiation-free technique, has the potential for monitoring the regional lung aeration and dynamic visualization of regional ventilation distribution at the bedside. Thus, EIT may be helpful in adaptive titration of PEEP and, consequently, could play an important role in the individualization of protective ventilation strategies. The reliability of EIT has already been proven in several studies [5-7]. The applications of EIT for selecting PEEP were recently proposed by Erlandsson and colleagues on morbidly obese patients [8] and Luepschen and colleagues in an animal study of lavage-induced lung failure [9].

A global inhomogeneity (GI) index based on EIT was recently developed to quantify the tidal volume distribution within the lung [10]. The aim of this study was to test the feasibility of optimizing PEEP with respect to ventilation homogeneity using the GI index. A retrospective study was performed and two other PEEP selection methods based on the analysis of lung mechanics, namely the maximum global dynamic compliance [11] and the compliance-volume curve method [12] were included for comparison.

## Materials and methods

### Patients and protocol

Ten sedated patients with healthy lungs (American Society of Anesthesiology (ASA) criteria I or ASA II; 7 male, 3 female; (mean ± standard deviation (SD)) age 30 ± 10 years; height 179 ± 8 cm; weight 77 ± 9 kg) were mechanically ventilated in volume-controlled mode (10 ml/kg body weight, ventilation frequency 12 min^-1^, inspiration:expiration ratio 1:1.5, fraction of inspired oxygen (FiO_2_) 1.0) for orthopedic surgery [10]. EIT measurement was performed before the surgical procedure. Exclusion criteria included age less than 18 years, pregnancy and lactation, history or clinical signs of lung disease, and any contraindication to the use of EIT (pacemaker, automatic implantable cardioverter defibrillator, and implantable pumps). The study was approved by the local ethics committee. Written informed consent was obtained from all patients prior to the study.

Anesthesia was induced by bolus injection of propofol and fentanyl, and was maintained by continuous infusion of propofol. Muscle relaxation was achieved with vecuronium bromide. After tracheal intubation (endotracheal tube inner diameter 7.0 for women and 8.0 for men) and confirmation of correct position of the tube, patients were mechanically ventilated with Evita4Lab (Dräger Medical, Lübeck, Germany). A standardized incremental PEEP trial [13] was performed before surgical procedure when all patients were in supine position. PEEP was increased from 0 to 28 mbar in steps of 2 mbar. Each PEEP level was maintained for 10 breaths. To standardize lung volume history, the maneuver was preceded by a zero end-expiratory pressure (ZEEP) ventilation phase lasting five minutes.

### Data collection and analysis

An EIT electrode belt, which carries 16 electrodes with a width of 40 mm, was placed around the thorax in the fifth intercostal space and one reference electrode was placed at the patients' abdomen. The EIT electrode belt was connected to an EIT monitor for online visualization (EIT Evaluation KIT 2, Dräger Medical, Lübeck, Germany). EIT data were generated by application of electrical alternating current (50 kHz, 5 mA peak-to-peak) in a sequential rotating process and measurement of the resulting surface potential differences between neighboring electrode pairs was performed. EIT images (each consists of 32 × 32 pixels) were subsequently generated with a newly developed reconstruction algorithm based on a modified 'finite element model' [3]. The images were continuously recorded at 20 Hz and stored. As electrocautery interferes with data acquisition of the prototype EIT device used in this study, the EIT electrode belt was removed shortly before surgery.

Airway pressure and gas flow rate were continuously recorded at 125 Hz. Volume was calculated as integral of gas flow rate after its correction for offset and drifts. These data were stored as ASCII files for synchronization with the EIT data. During the PEEP trial, we assumed that the respiratory signals reached their steady state after five breaths, because the step increase of PEEP levels was small. Data of five consecutive breathing cycles at the end of each PEEP level were pooled together in order to minimize the noise level in the signals.

The GI index was recently introduced by our group [10]. For every breathing cycle a so-called tidal image was generated. Each pixel of these tidal images represents the difference of impedance between end-inspiration and end-expiration. The median value of these tidal differences is calculated for the lung area in each tidal image. The sum of the absolute differences between the median value and every pixel value is considered to indicate the variation of the tidal volume distribution in the whole lung region. In order to make the GI index universal and secure inter-patient comparability, it is normalized by dividing it by the sum of the impedance values within the lung area:(1)

where *DI *denotes the value of the differential impedance in the tidal images; *DI*_*xy *_is the pixel in the identified lung area; *DI*_*lung *_are all pixels in the lung area under observation.

The identification of the lung area is a prerequisite for the GI calculation. A novel, EIT based lung area estimation method has been newly proposed [10,14]. In short, the areas found according to the functional EIT [5,15,16] by certain threshold [17] binarization are mirrored (left to right) and combined by means of a boolean "or"-operation. The cardiac-related area, which is distinguished in the frequency domain, is subsequently subtracted. As a result a quasi-symmetric left and right lung area is generated that includes all detectable lung area and that excludes the cardiac-related area.

The maximum global dynamic compliance is one of the most accepted parameters for setting PEEP [11,18,19]. It was included in the present study for comparison and compliance was calculated using the least-square-fit method [20].

Mols and colleagues suggested that the intra-tidal compliance-volume curve is able to indicate the ongoing recruitment and overdistension of alveoli in the lung [12]. Using the SLICE method, six consecutive volume-dependent compliances are obtained for a tidal breath [21]. The shapes of these curves are classified into mainly three groups: (1) a decrease in slope indicates overdistension; (2) an increase in slope indicates recruitment; (3) a quasi-horizontal compliance-volume curve indicates a suitable PEEP setting [12]. As comparison, the method, called compliance-volume curve method in the following, was also included in the present study.

### Statistical analysis

Statistical analysis was performed with the MATLAB software package (MATLAB 7.2 statistic toolbox, The MathWorks Inc., Natick, MA, USA). The Lilliefors test was used to evaluate the distribution of all data. For normally distributed data, results are presented as mean ± SD. Paired-sample t-test was applied in this case to assess the significance of differences in choosing PEEP levels for individuals (GI index vs. dynamic compliance; GI index vs. compliance-volume curve). A *P *value less than 0.05 was considered statistically significant. Due to the small amount of subjects in the study, significance levels were adjusted to maintain a statistical power above 80% in order to reduce the type II error. Furthermore, significance levels were corrected for multiple comparisons using Holm's sequential Bonferroni method. For not normally distributed data, results are expressed as median (interquartile range). Results were compared using the Bland-Altman analysis [22].

## Results

**Figure 1:**
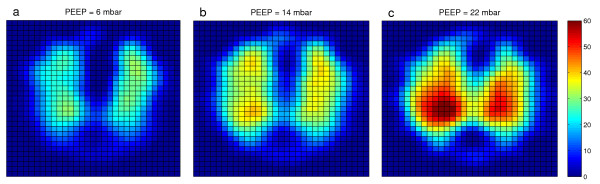
**Tidal ventilation distribution in EIT images at different PEEP levels**. **(a) **6 mbar. **(b) **14 mbar. **(c) **22 mbar. The tidal images were the differences of relative impedance between end-inspiration and end-expiration in electrical impedance tomography (EIT) images. High ventilated regions are marked in red, while low ventilated regions are marked in blue. PEEP = positive end-expiratory pressure.

Tidal volume distribution in EIT images (i.e. tidal images) at PEEP levels 6, 14 and 22 mbar are compared in Figure 1. With increased PEEP, the lung was further dilated.

**Figure 2:**
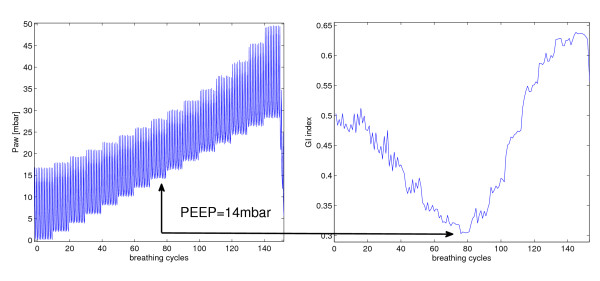
**A typical curve of (right) GI index of one patient (left) during a standardized PEEP trial**. The x axis displays the number of breathing cycles, counted once the maneuver started. A minimum value of the global inhomogeneity (GI) index indicated the optimal positive end-expiratory pressure (PEEP) with respect to ventilation homogeneity. Paw = pressure at airway opening.

In Figure 2, a typical relation between the GI value and PEEP is depicted. Starting at ZEEP, the GI index first decreased with the increase of PEEP indicating that ventilation was more homogenously distributed. A single minimum value of the GI index was found at a middle range of PEEP levels. With further increase in PEEP the GI index rose steadily (Figure 2). Such a curve with only single minimum value of the GI index was observed in every patient. At PEEP levels corresponding with the minimum GI index values (12.2 ± 4.6 mbar) the air is most homogenously distributed in the lungs.

**Figure 3:**
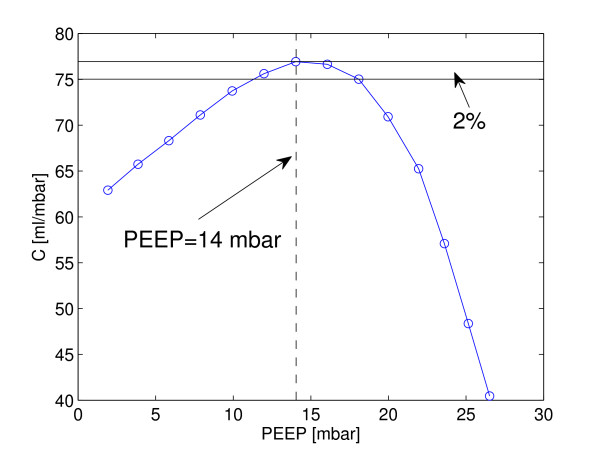
**Dynamic compliance calculated using the least-square-fit method for the same patient as in Figure 1**. Dashed-line indicates the optimized positive end-expiratory pressure (PEEP) level with respect to lung mechanics at 14 mbar where the compliance (C)-pressure curve reaches its maximum. A quasi-plateau phase in the curve is observed where the maximum relative change of compliance for 8 mbar pressure range is only 2% (relative to the maximum compliance value).

For comparison in Figure 3, the PEEP level is depicted for the same individual as in Figure 2 when the global dynamic compliance reached its maximum. A quasi-plateau phase in the compliance-pressure curve was found in every patient. In a range of 8 mbar (4 PEEP steps), the maximum relative change of compliance was only 2% (1%; in relation to maximum compliance).

**Figure 4:**
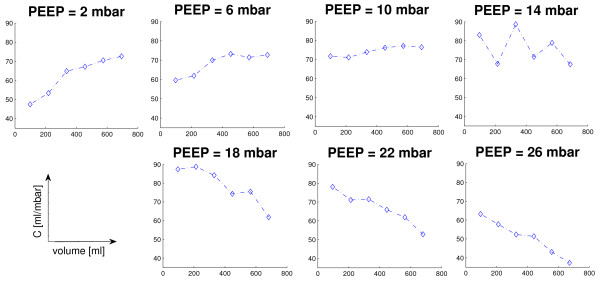
**The shape of intra-tidal dynamic compliance calculated with the SLICE method in the same patient as in Figures 1 and 2**. An upward slope indicates recruitment, a downward slope indicates overdistension and a quasi-horizontal shape indicates that neither the recruitment nor the overdistension effect is dominant. Positive end-expiratory pressure (PEEP) is optimized at 14 mbar in this patient according to the shape of the compliance (C)-volume curve.

According to the intra-tidal compliance-volume curves calculated with the SLICE method, another optimal PEEP level with respect to lung mechanics was obtained for every patient. Figure 4 shows typical intra-tidal compliance-volume curves in the same patient as in Figures 2 and 3. Positive slope (upwards direction) of the compliance-volume curves at a low PEEP indicates ongoing recruitment in inflation, while a negative slope (downwards direction) indicates overdistension of alveoli. PEEP is optimized when quasi-constant compliance within tidal breath is obtained [12].

**Figure 5:**
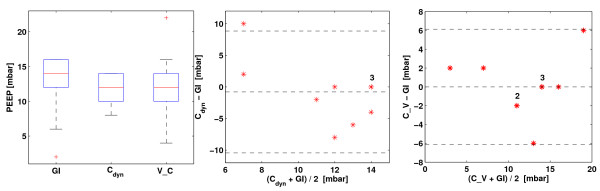
**Comparison of the optimal PEEP determined with the GI index, dynamic compliance and compliance-volume curve method**. Left = box plot. The boxes mark the quartiles while the whiskers extend from the box out to the most extreme data value within 1.5 times the interquartile range of the sample. Middle = Bland-Altman plot comparing global inhomogeneity (GI) and dynamic compliance method (C_dyn_). Right = Bland-Altman plot comparing GI and the intra-tidal compliance-volume curve calculated by the SLICE method (C_V). The numbers above the * indicate the number of overlapping results. The dashed line at the middle depicts the mean value of the whole data set. The other two dashed lines represent mean ± 1.96 times standard deviation.

Figure 5 shows the comparison of these methods in a box plot and Bland-Altman plots (GI index vs. dynamic compliance; GI index vs. compliance-volume curve). No significant differences in the results were found between the GI index method (12.2 ± 4.6 mbar) and the dynamic compliance method (11.4 ± 2.3 mbar, *P *> 0.6), or between the GI index and the compliance-volume method (12.2 ± 4.9 mbar, *P *> 0.6). Considering the quasi-plateau phases in compliance-pressure curves, the large differences between the results obtained with the GI index and the dynamic compliance method in some patients were explainable. No bias of results was observed in the Bland-Altman analysis.

## Discussion

In this study, we investigated the feasibility of our approach to optimize PEEP with respect to the homogeneity of pulmonary ventilation distribution using the GI index. In a previous study [10], we have demonstrated that the EIT-based GI index quantified the tidal volume distribution within the lung and showed good reliability and inter-patient comparability. Alveolar recruitment with less overdistension of the lung tissue would actually lead to a more homogeneous pulmonary air distribution. The feasibility of the GI index as a new tool in PEEP optimization was confirmed by the present retrospective study. The results were comparable with the global dynamic compliance method [11] and the intra-tidal compliance-volume curves produced by the SLICE method [12].

Although differences of air distribution in the lung can be observed in EIT images on a qualitative level (Figure 1), it is difficult to identify a superior PEEP level with respect to homogeneity of ventilation distribution. One reason is that the 'colourful' EIT-images only show the relative impedance values whereas the GI index quantifies the variation of the tidal volume distribution.

The results of all these three methods showed considerable inter-patient variation, which suggests the use of an individualized PEEP selection process. It has to be noted that the dynamic compliance and the compliance-volume curve method focus on the mechanics of the respiratory system, while the GI index focuses on a different aspect, namely the homogeneity of ventilation distribution. We have found no significant differences among the optimal PEEP values selected by these three methods, which indicates that homogeneity of air distribution in the lung has been somehow related to the global lung mechanics (at least to dynamic compliance). In the analysis of dynamic compliance, due to the quasi-plateau phase in the compliance-pressure curves (Figure 3), it is difficult to claim that the PEEP level where C = C_max _is superior to the level where C = C_max _× 98%. The difference between these two PEEP levels can be as large as 8 mbar. The PEEP selection using the compliance-volume curves is an enhancement of the dynamic compliance method. However, categorizing the compliance-volume curves is somehow complex and not intuitive. Therefore, another parameter to select PEEP in a different aspect is still needed. In addition, the GI index is superior to dynamic lung mechanics in spontaneously breathing patients where reliable lung mechanics are difficult to obtain.

The quasi-static P/V curve has also been used to individualize the setting of a proper PEEP level. But how to generate and analyze the P/V curve is still under intense debate [18]. To set PEEP at the lower inflection point plus 2 cmH_2_O was shown to be appropriate by Takeuchi and colleagues in a lavage-injured sheep ARDS model [23]. But there is no physiological interpretation to support it and the lower inflection point may be difficult to identify accurately [24], especially in patients with a wide distribution of opening pressures. New findings indicate that it may be better to derive PEEP from the upper inflection point of the deflation limb of the P/V curve [25]. In order to obtain quasi-static P/V curves, a normal ventilation process has to be interrupted in order to perform respiratory maneuvers, such as low-flow or super-syringe inflation. These maneuvers may be harmful to the patients due to hyper-inflation.

Besides using lung mechanics, there are other studies on open-lung PEEP selection using blood gas analysis [26-29] and imaging techniques [8,9,30], both of which are difficult to implement as a continuous bedside monitoring tool. Blood gas analysis provides a way to titrate PEEP but it is an invasive and discontinuous method. Recently, more and more studies on PEEP selection use imaging techniques. CT is the gold standard for assessment of tidal volume distribution in injured lungs [4]. Thus verification studies were normally based on CT examinations. However, CT is not an adequate method to monitor mechanical ventilation therapy due to radiation and the size of the device.

Using EIT instead of CT for bedside assessment of tidal volume distribution is a new trend. As the EIT images alone cannot be used objectively, quantifications were normally performed by calculating the ratio between different arbitrarily defined regions of interest [2,31-33]. Erlandsson and colleagues titrated PEEP to maintain a horizontal end-expiratory global relative impedance value, i.e. a stable end-expiratory lung volume, and claimed that such PEEP was optimal [8]. Although the partial pressure of oxygen (PaO_2_)/FiO_2 _ratio and compliance finally increased in these patients (not the maxima of PaO_2_/F_I_O_2_), there was no indication that these PEEP levels were optimal. Besides, how to identify the horizontal baseline has not explained in the literature. Luepschen and colleagues [9] modified the centre of gravity index from Frerichs and colleagues [16,34] to evaluate functional lung opening and overdistension of the lung tissue [9]. Unfortunately, we found more than one single minimum with their method on our data. This may be due to the differences in state of the lungs (healthy vs. lavage) or the differences in species (human vs. animal). Luepschen and colleagues also found that significant differences between dependent and non-dependent tidal volume loss and gain may reliably indicate recruitment and derecruitment of lung tissue [9]. But because they divided the EIT images into only two parts - a dorsal and a ventral - changes within each part were not detectable, leading to a coarse-grained method.

Unlike the global lung mechanics and static P/V curve, which are restricted to information integrating all lung regions [3], the GI index describes the inhomogeneity of tidal volume distribution in a cross-sectional lung plane where the EIT belt was placed in detail up to 32 × 32 regions. At the same time, with the help of a robust lung area determination method [10,14], the inhomogeneity analysis is restricted only to the lung region. Cardiac-related area and thorax area are excluded [10,14]. In addition, the GI index is a completely maneuver-free tool although in the present study an incremental PEEP trial was used. Without running the risk of inducing lung overinflation and ventilator-induced lung injury, PEEP may be adjusted according to the GI value. By adding small changes of PEEP, the gradient of the GI value indicates the direction of beneficial PEEP alteration.

Although a potential link between the homogeneity of air distribution in the lungs and dynamic respiratory mechanics is foreseen, a reference method to verify the homogeneity, such as CT, was missing in the study due to ethical reasons. Concrete evidence must be found to prove this relation or further validation with CT is needed before clinical application. Not only the lung mechanics but also the hemodynamic effect of PEEP may influence the decision of PEEP selection. It is reasonable to combine all these aspects (parameters) when titrating PEEP. The weights of different parameters are worth examining. Another drawback of the present study is that only patients with healthy lungs were recruited in the study. After this feasibility study, a further investigation on ALI/ARDS patients is essential. PEEP selection based on GI index or lung mechanics analysis may exhibit a different relation in patients suffering from severe respiratory insufficiency.

## Conclusions

In the present study, we found that a PEEP level, at which the lung was most homogenously ventilated, always existed during a standardized incremental PEEP trial. Such PEEP level is optimal with respect to ventilatory homogeneity and can be identified using the GI index. Moreover, the GI index may provide new insights into the relation between lung mechanics and tidal volume distribution. In further clinical evaluations it may be used to guide ventilator settings in combination with other aspects such as gas exchange and lung mechanics.

## Key messages

• The PEEP selection is a process depending on the individual properties of a patient and his or her disease state. Different aspects, such as blood gas, respiratory system mechanics and ventilatory homogeneity, need to be considered at the bedside.

• Evaluation of EIT data allows the incorporation of the patient's state of respiratory homogeneity into therapeutical decision-making at the bedside.

• It is feasible and reasonable to titrate the PEEP level with respect to ventilatory homogeneity based on EIT.

• Lung mechanics and tidal volume distribution are related. However, the relation may vary among different lung diseases.

## Abbreviations

ARDS: acute respiratory distress syndrome; ASA: american society of anesthesiology classification; CT: computed tomography; DI: the value of the differential impedance in the tidal images; DI_lung _: all pixels in the lung area under observation; DI_xy _: the pixel in the identified lung area; EIT: electrical impedance tomography; GI: global inhomogeneity; PaO_2 _: partial pressure of arterial oxygen; PEEP: positive end-expiratory pressure; P/V: pressure-volume curve; SD: standard deviation; ZEEP: zero end-expiratory pressure.

## Competing interests

The authors declare that they have no competing interests.

## Authors' contributions

ZZ designed the study, analyzed the data and drafted the manuscript. DS carried out the data measurement. IF revised the manuscript critically. JG gave valuable advices and contributed to writing. KM contributed to study design, data analysis and writing. All authors read and approved the final manuscript.
